# Genome-Wide Analysis of Gene Regulatory Networks of the FVE-HDA6-FLD Complex in *Arabidopsis*

**DOI:** 10.3389/fpls.2016.00555

**Published:** 2016-04-28

**Authors:** Chun-Wei Yu, Kao-Yuan Chang, Keqiang Wu

**Affiliations:** Institute of Plant Biology, College of Life Science, National Taiwan UniversityTaipei, Taiwan

**Keywords:** MSIL, FVE, histone deacetylase, HDA6, flowering, *Arabidopsis*

## Abstract

FVE/MSI4 is a homolog of the mammalian RbAp48 protein. We found that FVE regulates flowering time by repressing *FLC* through decreasing histone H3K4 trimethylation and H3 acetylation. Furthermore, FVE interacts with the histone deacetylase HDA6 and the histone demethylase FLD, suggesting that these proteins may form a protein complex to regulate flowering time. To further investigate the function of the FVE-FLD-HDA6 complex, we compared the gene expression profiles of *fve*, *fld*, and *hda6* mutant plants by using RNA-seq analysis. Among the mis-regulated genes found in *fve* plants, 51.8 and 36.5% of them were also mis-regulated in *fld* and *hda6* plants, respectively, suggesting that FVE, HDA6, and FLD may regulate the gene expression in the same developmental processes in *Arabidopsis*. Gene ontology analysis revealed that among 383 genes co-regulated by FVE, HDA6, and FLD, 15.6% of them are involved in transcription, 8.2% in RNA metabolic process, 7.7% in response to abiotic stress, and 6.3% in hormone stimulus. Taken together, these results indicate that HDA6, FVE, and FLD co-regulate the gene expression in multiple development processes and pathways.

## Introduction

MSI1-like WD40 repeat (MSIL) proteins are subunits of many protein complexes controlling chromatin dynamics (Hennig et al., 2005). MSI1 was first found in a screen for multicopy suppressors of the *ira1* mutation in yeast and was therefore termed MSI1. Most MSIL proteins contain seven WD40 domains (Smith et al., 1999; Van Nocker and Ludwig, 2003). The number and sequence of intervening amino acids between individual WD40 repeats are different in MSIL proteins compared with other WD40 repeat proteins (Vermaak et al., 1999). In *Arabidopsis*, three clades are represented by MSI1, MSI2, and MSI3, as well as MSI4/FVE and MSI5 (Hennig et al., 2005). The function of MSIL proteins in plants has also been characterized. Loss of *MSI1* function in *Arabidopsis* causes seed abortion (Köhler et al., 2003; Ausín et al., 2004; Guitton et al., 2004; Kim et al., 2004). *FVE/MSI4* was identified by screening for late flowering mutants in *Arabidopsis* (Koornneef et al., 1991; Ausín et al., 2004). It was found that FVE/MSI4 is required for transcriptional repression of *FLOWERING LOCUS C (FLC)* encoding a repressor of the transition from vegetative to reproductive development in *Arabidopsis.*

In plants, the transition from a vegetative to a reproductive phase is critical to reproductive success and is genetically controlled by a network of flowering genes (He, 2009). In *Arabidopsis*, multiple genetic pathways act in response to developmental cues and environmental signals to control the floral transition, including vernalization, autonomous, photoperiod, and gibberellin-dependent pathways (Mouradov et al., 2002; Boss et al., 2004). *FLC* encodes an MADS-box transcription factor and plays a central role in flowering-time regulation in *Arabidopsis* (Michaels and Amasino, 1999; Sheldon et al., 1999). In winter annual plants, *FRIGIDA (FRI)* and a *FRI* relative *FRI LIKE 1 (FRL1)* act to up-regulate the expression of *FLC*, whereas a non-functional *FRI* allele (such as Col ecotype) contributes to the early flowering phenotype (Johanson et al., 2000; Michaels et al., 2003).

The vernalization and autonomous pathways converge on and repress the expression of *FLC*. Prolonged cold exposure (vernalization) leads to a series of repressive histone modifications in *FLC* chromatin, including histone deacetylation, H3K4 demethylation, as well as H3K9 and H3K27 di- and tri-methylation (Bastow et al., 2004; Sung and Amasino, 2004; Sung et al., 2006; Finnegan and Dennis, 2007). Genetic screening identified a number of autonomous pathway genes including *FVE*, *FLD*, *LD*, *FLK*, *FY*, *FCA*, and *FPA* (Koornneef et al., 1998; Simpson, 2004). Mutations in these loci result in late flowering in both long day and short day photoperiods (Koornneef et al., 1998; Simpson, 2004). RNA processing plays a crucial role in the autonomous pathway. The function of FCA, FPA, and FY involves a set of long non-coding antisense transcripts termed *COOLAIR* at the *FLC* locus (Hepworth and Dean, 2015). Both FCA and FPA are RNA-recognition motif (RRM)-type RNA-binding proteins and they function partially redundantly to control alternative splicing and 3′-end processing of mRNAs (Hornyik et al., 2010). FY is a cleavage and poly(A) specificity factor component. FCA and FPA act with FY and the cleavage stimulation factors CstF64 and CstF77 to promote the choice of the proximal poly(A) site (Liu et al., 2010). This process is also regulated by the activity of the core spliceosome component PRP8 and CYCLIN DEPENDENT KINASE GROUP C2 (CDKC2; Marquardt et al., 2014; Wang et al., 2014). In addition, other proteins such as GRP7 and PRP39 involved in various aspects of RNA metabolism have also been associated with the autonomous pathway (Wang et al., 2007; Streitner et al., 2008).

More recent studies indicated that histone modifications are also involved in the autonomous pathway of flowering. *FLD* encoding a Lysine Specific Demethylase1 (LSD1) type histone demethylase is involved in the histone H3 lysine 4 demethylation (He et al., 2003; Liu et al., 2007). The histone deacetylase HDA6 regulates flowering time by directly interacting with FLD (Yu et al., 2011). Increased levels of histone H3 acetylation and H3K4 trimethylation at *FLC* were found in both *hda6* and *fld* mutant plants, suggesting that both HDA6 and FLD are involved in *FLC* repression by histone deacetylation and demethylation. Analysis of *fld* mutants also suggested a positive feedback mechanism coupling histone methylation with COOLAIR splicing and polyadenylation (Liu et al., 2010; Marquardt et al., 2014). Alternative processing of COOLAIR leads to histone methylation changes of *FLC*, which provides a positive feedback loop reinforcing splicing and chromatin modification outcomes. Furthermore, HDA6 was also shown to associate with FVE/MSI4 in repression of *FLC* expression (Gu et al., 2011). Collectively, these data suggested that HDA6 may form a HDAC complex with FVE and FLD to regulate gene expression in control of flowering time.

In this study, we further investigated the function of FVE and its interaction with HDA6 and FLD. We showed that FVE regulates flowering time by repressing *FLC* through decreasing H3K4 trimethylation and H3 acetylation. Furthermore, transcriptome analysis indicated that FVE, HDA6, and FLD co-regulate the gene expression involved in cell wall-loosening, transport, transcription, and hormone signaling in *Arabidopsis*.

## Materials and Methods

### Plant Materials

*Arabidopsis* plants were grown under long day (16 h light, 8 h dark) or short day (8 h light, 16 h dark) conditions. *hda6-6 (axe1-5)* is a *hda6* mutant carrying a point mutation on *HDA6* splicing site (Murfett et al., 2001), whereas *fve-4* has a point mutation resulting an early stop in translation of *FVE* (Ausín et al., 2004; Kim et al., 2004). *fld-6* is a T-DNA insertion mutant line (SAIL_642_C05) carrying a T-DNA insertion in the second exon of *FLD* (Yu et al., 2011).

### RNA Extraction and Quantitative RT-PCR Analysis

Total RNA was isolated with the TRIZOL Reagent (Invitrogen) according to the manufacture’s protocol. To synthesize cDNA, 2 microgram of total RNA was used to synthesize cDNA by MMLV Reverse Transcriptase (Promega). Real-time PCR was performed by using iQ SYBR Green Supermix solution (Bio-Rad). The gene specific primers used for real-time RT-PCR are listed in Supplementary Table S12. Each sample was quantified at least in triplicates and normalized using *Ubiquitin10 (UBQ)* as an internal control.

### Chromatin Immunoprecipitation Assays

Chromatin immunoprecipitation (ChIP) assays were performed as described (Yu et al., 2011). The chromatin was sheared to an average length of 500 bp by sonication for immunoprecipitation. The following antibodies were used: anti-acetylated histone H3K9K14 (Millipore; Catalog no. 06-599), anti-tri-methylated histone H3K4 (Millipore; Catalog no. 04-745), and anti-tri-methylated histone H3K27 (Millipore; Catalog no. 17-622). The DNA cross-linked to immunoprecipitated proteins was reversed and recovered by Phenol: Chloroform: Isoamyl Alcohol (25:24:1) purification. Then, the DNA was analyzed by real-time PCR using specific primers (Supplementary Table S12).

### Bimolecular Fluorescence Complementation (BiFC) Assays

To generate the constructs for BiFC assays, full-length cDNA fragments of HDA6, FVE, and FLD were PCR-amplified and cloned into the pCR8/GW/TOPO (Invitrogen) vectors, and then recombined into the YN (pEarleyGate201-YN) and YC (pEarleyGate202-YC) Vectors (Lu et al., 2010). Constructed vectors were transiently transformed into tobacco (*Nicotiana benthamiana*) leaves. Transfected leaves were then examined using a TCS SP5 (Leica) Confocal Spectral Microscope Imaging System.

### Genome-Wide mRNA Sequencing

Total RNA was prepared using a standard TRIZOL Reagent extraction method from 0.2 to 0.3 g of 2-week-old *Arabidopsis* plants. Poly-A containing mRNA molecules were purified using poly-T oligo-attached magnetic beads. Then, cDNA was synthesized by using random Hexamer priming. The second-strand was generated to create double-stranded cDNA. cDNA templates were purified by using the Qiagen kit followed by end repair, poly A tailing and adaptor connection. Libraries were sequenced using the Illumina HiSeq^TM^ 2500. Illumina’s CASAVA pipeline (Version 1.8) was used to produces FASTQ files. More than 20 million clean reads were obtained in each sample. All clean reads were mapped to the TAIR10 genome^1^. RPKM (reads per kilobase of exon model per million mapped reads; Mortazavi et al., 2008) values were computed based on these mapped reads using RackJ^2^, and student *T*-tests were carried out based on RPKM values. Genes were consider as significantly differentially expressed with a *p*-value < 0.05 and relative change threshold of twofold. GO terms and functionally clusters were analyzed with the DAVID Web tools (Huang et al., 2009). Both heatmap and scatter plot analysis were done in R project (Severin et al., 2010). The functional clusters enrichment analysis was calculated by comparing the whole *Arabidopsis* genome, and the highest classification was selected for clustering.

For Col and *hda6-6*, three biological repeats were performed and the data are consistent for each genotype (Supplementary Figure S5). For *fld-6* and *fve-4*, one library was analyzed for each genotype and qRT-PCR was performed to validate RNA-seq data (Supplementary Figure S4). The RNA-seq data were deposited in GeneBank (Accession number: GSE78946).

## Results

### *hda6-6* and *fve-4* Mutants Display Delayed Flowering

**FIGURE 1:**
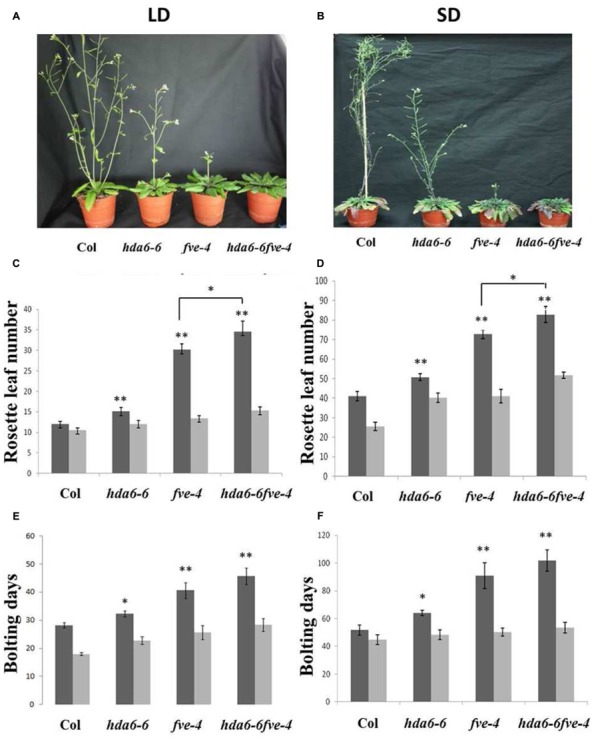
**Delayed flowering phenotypes of *hda6-6*, *fve-4* and *hda6-6fve-4* plants. (A,B)** *hda6-6*, *fve-4*, and *hda6-6fve-4* plants grown under long day (LD) and short day (SD) conditions compared with Col wild type. **(C–F)** Rosette leaf numbers and bolting days of Col, *hda6-6*, *fve-4*, and *hda6-6fve-4* plants grown under LD and SD without vernalization (black columns) or with 45 days vernalization (gray columns). At least 20 plants were scored for each line. Error bars indicate SD. *P < 0.05, **P < 0.01 (*t*-test).

*hda6-6 (axe1-5)* is a *hda6* mutant carrying a point mutation on *HDA6* splicing site (Murfett et al., 2001; Blevins et al., 2014), whereas *fve-4* has a point mutation resulting an early stop in translation of *FVE* (Kim et al., 2004). To investigate the genetic interaction between *HDA6* and *FVE*, *hda6-6 fve-4* double mutants were generated by crossing *hda6-6* and *fve-4* mutants. Both *hda6-6* and *fve-4* mutant plants displayed later flowering phenotypes under long-day (LD, 16 h light and 8 h dark) and short-day (SD, 8 h light and 16 h dark) conditions (**Figures 1A,B**), as measured by the days of bolting and the rosette leaf numbers at flowering (**Figures 1C–F**). Compared to *hda6-6* and *fve-4* plants, the flowering time of *hda6-6 fve-4* plants was further delayed under both LD and SD (**Figure 1**). The delay in flowering time of *hda6-6*, *fve-4*, and *hda6-6 fve-4* mutants was completely corrected by 45 days of vernalization at 4°C (**Figures 1C–F**), supporting that both FVE and HDA6 are involved in the autonomous pathway of flowering transition.

**FIGURE 2:**
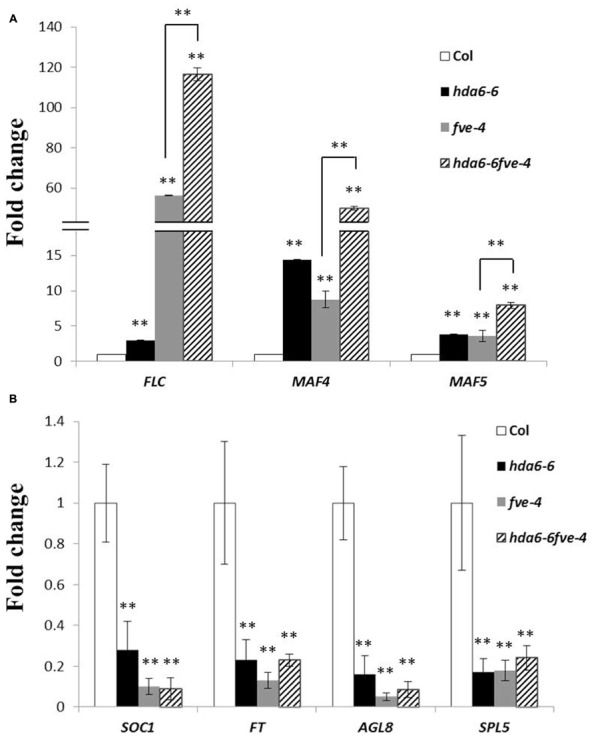
**Expression of flowering repressor and activator genes. (A)** Real-time RT-PCR analysis of the expression of *FLC*, *MAF4*, and *MAF5* in Col wild type, *hda6-6*, *fve-4*, and *hda6-6fve-4* plants grown under LD conditions for 20 days. The values shown are means ± SD. **(B)** Real-time RT-PCR analysis of the expression of *SOC1*, *FT*, *AGL8*, and *SPL5* in Col, *hda6-6*, *fve-4*, and *hda6-6fve-4* plants grown under LD conditions for 20 days. The values shown are means ± SD. ** Double asterisks denote statistical significance *P* < 0.01 (*t*-test).

We compared the expression of *FLC, MAF4*, and *MAF5* in *hda6-6*, *fve-4*, and *hda6-6 fve-4* plants by qRT-PCR. As shown in **Figure 2A**, *FLC*, *MAF4*, and *MAF5* were up-regulated in *hda6-6*, *fve-4*, and *hda6-6fve-4* plants compared with wild type plants. In contrast, the transcript levels of two downstream flowering integrators, *FT* and *SOC1*, as well as two flowering activators, *AGL8* and *SPL5*, were decreased in *hda6-6*, *fve-4*, and *hda6-6 fve-4* plants (**Figure 2B**).

### Histone H3 Acetylation and H3K4 Trimethylation Levels of *FLC* are Increased in *hda6-6, fve-4*, and *hda6-6 fve-4* Plants

**FIGURE 3:**
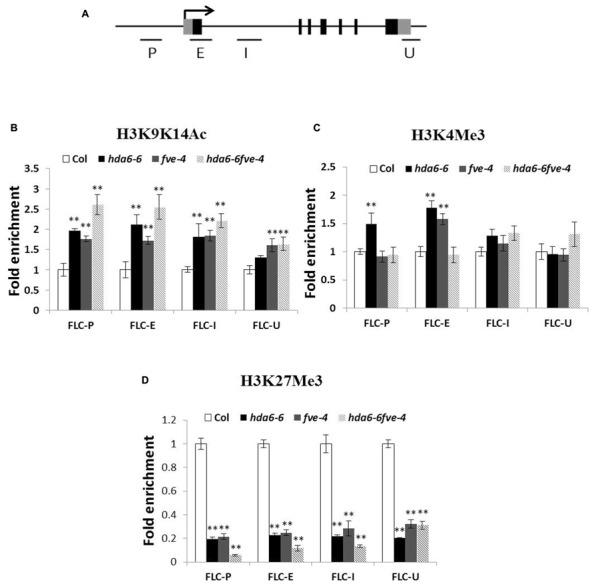
**Histone modification levels of *FLC* chromatin in *hda6-6*, *fve-4*, and *hda6-6fve-4* mutants. (A)** Schematic structure of genomic sequences of *FLC* and the regions examined by ChIP. **(B–D)** Relative levels of H3K9K14Ac **(B)**, H3K4Me3 **(C)**, and H3K27Me3 **(D)** of *FLC* in Col, *hda6-6*, *fve-4*, and *hda6-6 fve-4* seedlings grown under LD condition for 10 days. ** Double asterisks denote statistical significance *P* < 0.01 (*t*-test).

To analyze whether the high expression of *FLC* in mutants is related to histone modifications in chromatin, ChIP assays were used to analyze the histone H3 acetylation level. As shown in **Figures 3A,B**, hyperacetylation of histone H3 was found in the promoter (P), first exon (E) and intron (I) regions of *FLC* in *hda6-6*, *fve-4*, and *hda6-6 fve-4* plants. ChIP assays were also used to analyze the histone H3K4 methylation level of *FLC*. As shown in **Figure 3C**, hypermethylation of histone H3K4 was also found in the promoter, first exon and intron regions of *FLC* in the mutants.

We further analyzed the histone H3K27 methylation level of *FLC* in *hda6-6*, *fve-4*, and *hda6-6 fve-4* plants. As shown in **Figure 3D**, hypomethylation of histone H3K27 was found in the promoter, first exon and intron as well as 3′-UTR regions of *FLC.* These results suggested that HDA6 and FVE regulate the expression of *FLC* by affecting chromatin acetylation and methylation.

### Genome-Wide Transcription Analysis of *fve*, *fld*, and *hda6* Mutants

**FIGURE 4:**
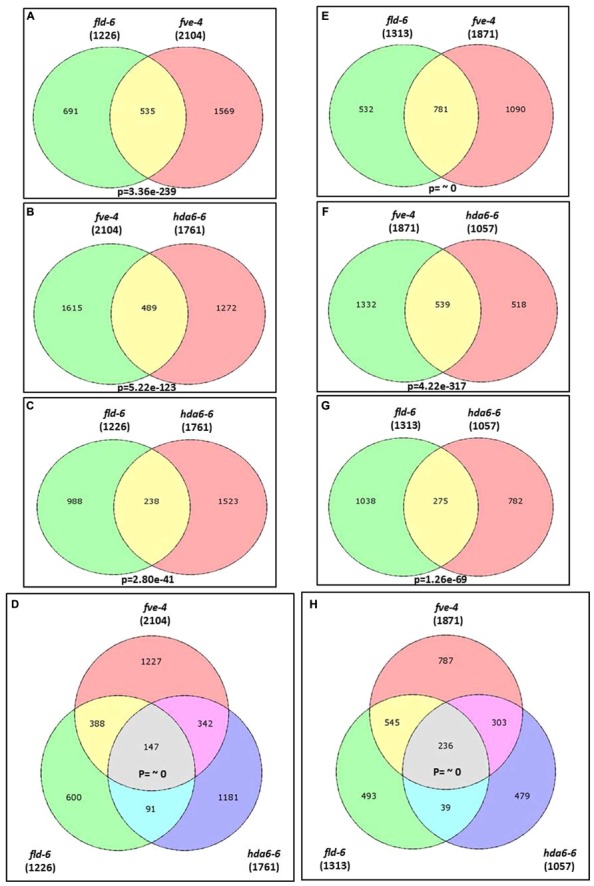
**Transcriptome profiles of *fve-4*, *fld-6* and *hda6-6* mutants. (A–D)** Venn diagrams showing the number of genes up-regulated (>twofold, *p*-value < 0.05) in *fve-4*, *fld-6*, and *hda6-6* mutants by using RNA-sequencing analysis. **(E–H)**. The number of genes down-regulated (<twofold, *p*-value < 0.05) in *fve-4*, *fld-6*, and *hda6-6* mutants. Numbers in parentheses represent the total numbers of up- or down-regulated genes in the respective mutants. The *P*-value of the Venn diagram **(A–C** and **E–G)** was calculated using the hypergeometric distribution. **(D,H)** Simulation-based *P*-values were calculated via R project.

Previous studies indicated that the *Arabidopsis* HDA6 is associated with FLD and FVE, forming HDAC complexes that control flowering time (Gu et al., 2011; Yu et al., 2011). We found that FVE can also interact with FLD by using bimolecular fluorescence complementation (BiFC) analysis (Supplementary Figure S1). These data suggested that FVE, FLD, and HDA6 may form a protein complex to regulate gene expression. To further study the function of FVE, FLD, and HDA6 in *Arabidopsis*, we compared the transcriptome of *fve-4*, *fld-6*, and *hda6-6* mutants with wild type by RNA-sequencing. Total RNA were extracted from 14-day old plants grown under LD conditions. Genes with ≥ twofold increased or decreased expression and *P*-value ≤ 0.05 were considered to have significant expression differences. Differentially expressed genes identified in the mutants are listed in Supplementary Tables S3–S8. Compared with Col wild type, 1761 (62.5%) genes were up-regulated and 1057 (37.5%) genes were down-regulated in *hda6-6*; 2104 (52.9%) genes were up-regulated and 1871 (47.1%) genes were down-regulated in *fve-4*; whereas 1226 (48.3%) genes were up-regulated and 1313 (51.7%) genes were down-regulated in *fld-6* (**Figure 4**).

**FIGURE 5:**
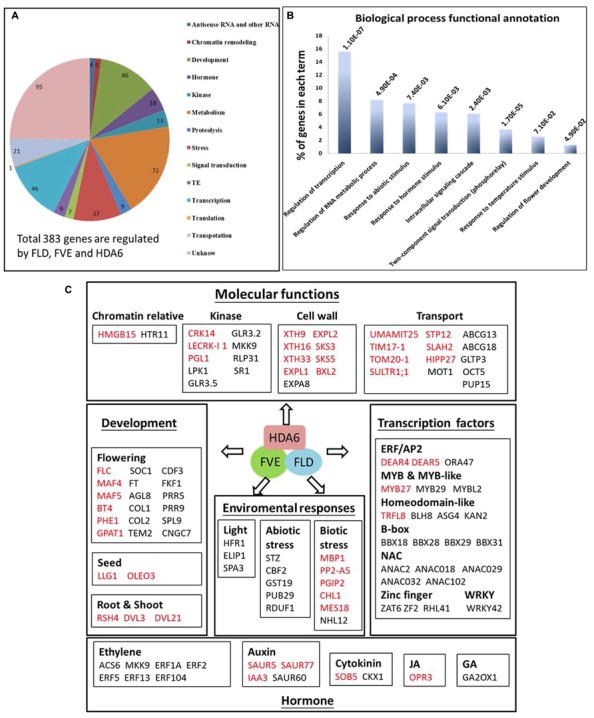
**Functional analysis of co-regulate genes in *fve-4, fld-6*, and *hda6-6* mutants. (A)** Gene Ontology classification of FVE, FLD, and HDA6 co-regulated genes. **(B)** DAVID functional clustering of the highly enriched GO terms in FVE-FLD-HDA6 co-regulated genes. **(C)** Representative FVE-FLD-HDA6 co-regulated genes with known functions in various molecular functions including development, enviromental responses, transcription regulation, and hormone responses. Genes repressed and activated by FVE-FLD-HDA6 are in red and black, respectively.

Among the mis-regulated genes found in *fve-4*, 51.8 and 36.5% of them were also mis-regulated in *fld-6* and *hda6-6*, respectively (**Figure 4**), suggesting that FVE, HDA6, and FLD may regulate the gene expression involved in the same developmental pathways. Among 383 genes co-regulated by FVE, HDA6, and FLD, 147 genes were up-regulated (**Figure 4D**), whereas 236 genes were down-regulated (**Figure 4H**) in the mutants. Next, we performed the functionally clustered and the gene ontology (GO) analysis of these co-regulated genes by using the DAVID (The Database for Annotation, Visualization, and Integrated Discovery) resource (Huang et al., 2009). The predicted gene functions include chromatin remodeling, transcription, development, phosphorylation, metabolism, proteolysis, stress response, transport, and others (**Figure 5A**). In addition, GO analysis revealed that the major functions of these co-regulated genes are involved in transcription (15.6%), RNA metabolic process (8.2%), response to abiotic stress (7.7%), response to hormone stimulus (6.3%), as well as intracellular signaling cascade (6.1%; **Figure 5B**).

### FVE, FLD, and HDA6 Co-regulate Cell Wall-Loosening, Transport, Flowering and Hormone Related Genes

From our transcriptome analysis, we found that genes related to cell wall-loosening, transport, flowering, and hormones are co-regulated by FVE, FLD, and HDA6 (**Figure 5C**). A large number of genes that are involved in cellular processes such as cell wall and transport are co-regulated by FVE, FLD, and HDA6. *SMALL AUXIN UP-RNA (SAUR)* genes play a significant role in promotes cell expansion (Cosgrove, 1998; Spartz et al., 2014). In addition, xyloglucan endotransglycosylases/hydrolases (XTHs) and expansins (EXPs) are two groups of enzymes important in cell wall loosening and cell expansion (Li et al., 2003; Van Sandt et al., 2007). A number of genes encoding SAURs, XTHs, and EXPs were repressed by FVE, FLD and HDA6 (**Figure 5C** and Supplementary Table S1), suggesting that the FVE-FLD-HDA6 module plays a role in stabilizing the cell wall.

The transition of nutrients and metals play essential roles in physiological processes including plant growth, nutrition, signal transduction, and development (Hall and Williams, 2003). In *Arabidopsis* genome, approximately 5% of genes (more than 800 genes) encode membrane transport proteins (Mäser et al., 2001). Our RNA-sequencing analysis revealed that at least 21 genes related to nutrient and metal transport including *UMAMIT25*, *TIM17-1*, *TOM20-1*, *SULTR1;1*, *STP12*, *HIPP27*, *ABCG13*, *ABCG18*, *GLTP3*, *MOT1*, *OCT5*, and *PUP15* were affected in the mutants (Supplementary Tables S1 and S2; **Figure 5C**).

In addition, several genes related to hormone biosynthesis and signaling were also mis-regulated in *fve-4*, *fld-6*, and *hda6-6* mutants (**Figure 5**). The expression of *ACS6* encoding 1-Aminocyclopropane-1-Carboxylic Acid (ACC) synthase in ethylene biosynthesis was down-regulated in *fve-4*, *fld-6*, and *hda6-6* mutants (Supplementary Table S2; **Figure 5C**). Furthermore, the expression of *MKK9*, *ERF2*, and *ERF5* involved in ethylene signaling was all reduced in the mutants. These data suggest that FVE, FLD, and HDA6 may co-regulate the gene expression involved in ethylene biosynthesis and signaling.

### FVE Regulates Gene Expression Involved in RNA Processing and Temperature Stimulus

**FIGURE 6:**
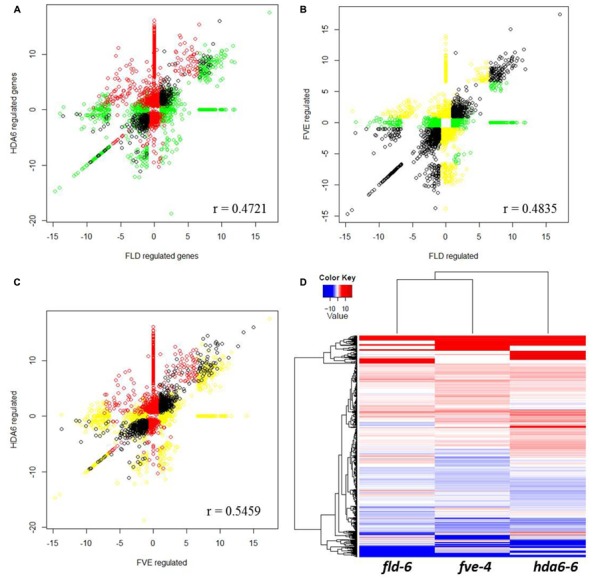
**Scatterplot analysis of FVE, FLD, and HDA6 regulated genes. (A–C)** Correlation by scatterplot of log2 fold change values between HDA6 and FLD regulated genes **(A)**, FVE and FLD regulated genes **(B)** and HDA6 and FVE regulated genes **(C)**. Correlation coefficient was calculated and shown in figure. **(D)** Transcriptional profiles comparisons represented as a heatmap to highlight up-regulated (red) and down-regulated (blue) genes in *fve-4*, *fld-6*, and *hda6-6* mutants.

We further performed the scatterplot and heat map analysis of FVE, FLD, and HDA6 regulated genes (**Figure 6**). Although FVE, FLD, and HDA6 co-regulate a large number of genes (**Figures 6A–C**, black circle), they also have specialized functions (red, yellow, and green circle in **Figures 6A–C**). The heat map analysis showed the similar patterns among *fve-4*, *fld-6*, and *hda6-6* regulated genes, but several groups of genes were also independently regulated by FVE, FLD, or HDA6 (**Figure 6D**).

To gain further insight into the individual biological roles of FVE, FLD, and HDA6, we selected the up-regulated genes in each mutant for further analysis (Supplementary Figures S3A–F; Supplementary Tables S9–S11). In the *fve-4* mutant, a high enrichment in terms related to RNA processing was observed (Supplementary Figure S3A). Also, genes responding to temperature stimulus were also enriched, consisting with the proposed function of FVE in cold stress regulation (Kim et al., 2004). A high proportion of genes involved in ribonucleoprotein complex biogenesis, non-coding RNA (ncRNA) metabolic process, ribosome biogenesis, as well as a cell wall organization were identified, suggesting the potential role of FVE in these processes.

The GO analysis also highlighted the potential role of HDA6 in oxidation-reduction, defense response, phosphorylation, intracellular signaling cascade, cell death, as well as cell wall organization (Supplementary Figure S3C). High proportion genes are found in oxidation-reduction, defense response, and cell death, consistent with previous studies showing that HDA6 is required for abiotic stress and plant defense (Chen et al., 2010; Zhu et al., 2011). Interesting, 57 genes related to phosphorylation were exclusive to the *hda6* mutant (*hda6-6*; Supplementary Figure S3C; Supplementary Table S11). Also, a large number of transposons were specifically up-regulated in the *hda6-6* mutant (Supplementary Figures S2 and S3F; Supplementary Table S11), supporting the role of HDA6 in controlling the stability of transposons.

## Discussion

MSIL proteins were found in all eukaryotes but seem to be absent in prokaryotes (Hennig et al., 2005). In mammalian and yeast cells, MSIL proteins are subunits of many protein complexes controlling chromatin assembly, DNA damage repair, and stress-sensing signaling pathways (Hennig et al., 2005; Yang et al., 2013). Most of the MSIL proteins contain seven WD40 repeats and are believed to form a β-propeller fold structure which is important for protein-protein interactions (Smith et al., 1999).

In plants, *Arabidopsis thaliana* contains five MSIL proteins (MSI1 to MSI5), while rice (*Oryza sativa*) and *Zea mays* have three MSIL proteins. The *Arabidopsis* MSIL proteins can be classify into three main clades, including MSI1, MSI2/MSI3, and MSI4/MSI5 (Hennig et al., 2005). *Arabidopsis* MSI1 is a component of the MEA/FIE polycomb group complex, and loss of function of *MSI1* causes seed abortion, indicating that MSI1 is required for seed development (Köhler et al., 2003). FVE/MSI4 has been shown to repress expression of the central floral repressor *FLC* and several cold-responsive genes, including *COR15a* and *COR47* in *Arabidopsis* (Ausín et al., 2004; Kim et al., 2004). More recently, MSI5 was found to acts in partial redundancy with MSI4/FVE to silence *FLC* by associating with HDA6 (Gu et al., 2011; Yu et al., 2011).

The transition from vegetative to reproductive development in *Arabidopsis* is controlled by several independent pathways. *FLC* is a major repressor in flowering and is epigenetically regulated in response to both endogenous and environmental cues (Michaels and Amasino, 2001; He, 2009). The autonomous pathway genes including *FLD*, *HDA6*, and *FVE* repress the *FLC* expression through histone modifications. FLD is a lysine-specific demethylase 1-type histone demethylase involved in the removes methyl groups from mono- and dimethylated histone H3K4 (Jiang et al., 2007; Liu et al., 2007). HDA6 is a histone deacetylase catalyzing the removal of acetyl groups from lysine residues of histone. We found that FVE can interact with both FLD and HDA6, indicating that these proteins may be part of the same protein complex. Similar to *hda6* and *fld* mutants (Yu et al., 2011), increased levels of histone H3 acetylation and H3K4 trimethylation at *FLC* were also found in *fve* plants. These data support a scenario in which *FLC* repression associated with histone deacetylation and H3K4 demethylation in the autonomous pathway is mediated by the interaction of FVE with HDA6 and FLD.

*FLOWERING LOCUS C* is epistatic to *FLD* regarding flowering time, since the *flc fld* double mutant flowers as late as a *flc* single mutant (He et al., 2003). By contrast, the *hda6-6* mutant delays flowering even in an *flc-3* mutant background (Yu et al., 2011), suggesting that HDA6 may control flowering independent of FLC regulation. The flowering time phenotypes in *fld* and *fve* mutants are much stronger compared to *had6* mutants. Furthermore, *hda6 fld* and *hda6 fve* double mutants are later flowering compared to *fld* and *fve* single mutants, supporting non-redundant roles of HDA6 and FVE/FLD. A likely explanation may be functional redundancy between members of the HDAC protein family. Indeed, another HDAC, HDA5, was also found to be involved in flowering by interacting with FVE and FLD (Luo et al., 2015).

Histone H3K27 methylation is regulated by the Polycomb Repressor Complex 1 (PRC1) and PRC2 (Dellino et al., 2004; Francis et al., 2004; King et al., 2005). PRC2 acts as a histone methyl-transferase that catalyzes tri-methylation of histone H3K27, whereas PRC1 inhibits transcription and blocks remodeling of the target nucleosomes by binding the H3K27me3 marks. *Drosophila* PRC2 has been shown to be associated with histone deacetylases, suggesting that histone deacetylation is linked to the PRC2-mediated gene repression suppression (Tie et al., 2001). Recent studies showed that MSI4 represses *FLC* expression through its association with a PRC2 complex in *Arabidopsis* (Frolov and Dyson, 2004; Pazhouhandeh et al., 2011). In this study, hypomethylation of histone H3K27 was found in *FLC* chromatin of both *fve* and *hda6* mutants. Taken together, these results support that the FLD-FVE-HDA6 complex may act with the PRC2 complex to silence *FLC* in flowering regulation.

Among the mis-regulated genes found in *fve-4*, 51.8 and 36.5% were also mis-regulated in *fld-6* and *hda6-6*, respectively. The overlap among the mis-regulated genes in these mutants suggested that FVE, FLD, and HDA6 may functional together to control multiple plant developmental pathways. The SAUR19-24 subfamily of SAUR proteins play a significant role in promoting cell expansion (Cosgrove, 1998; Spartz et al., 2014). In addition, EXPs and XTHs were proposed to act as cell wall-loosening agents (Fry et al., 1992; Nishitani and Tominaga, 1992; Cosgrove, 2000). We found that many members of *SAUR*, *EXP*, and *XTH* genes were repressed by FVE, FLD, and HDA6 (Supplementary Tables S1, S5, and S7; **Figure 5C**), suggesting that the FLD-FVE-HDA6 complex is involved in the regulation of cell expansion.

ABCG transporters are required for suberin and pollen wall extracellular barriers in *Arabidopsis* (Yadav et al., 2014). Five *Arabidopsis* ABCG transporters including ABCG2, ABCG6, and ABCG20 are required for synthesis of an effective suberin barrier in roots and seed coats, whereas ABCG1 and ABCG16 are required for the development of pollen wall (Yadav et al., 2014). We found that the expression of *ABCG13* and *ABCG18* was reduced in *fve-4*, *fld-6*, and *hda6-6* mutants (Supplementary Table S2). In addition to *ABCG13* and *ABCG18*, our RNA-sequencing analysis revealed that at least 21 genes related to nutrient and metal transport were also affected in the mutants (Supplementary Tables S1 and S2, **Figure 5C**), supporting that the FLD-FVE-HDA6 complex is required for controlling transport in plants. Further research is required to reveal the molecular mechanism of the involvement of the FLD-FVE-HDA6 complex in the regulation cell expansion and transport.

## Author Contributions

C-WY and KW conceived this project and designed all research. C-WY and K-YC performed the research. C-WY and KW analyzed data and wrote the article.

## Conflict of Interest Statement

The authors declare that the research was conducted in the absence of any commercial or financial relationships that could be construed as a potential conflict of interest.

## Footnotes

1 http://www.arabidopsis.org/

2 http://rackj.sourceforge.net/
