# Supplementary figures and images for: Genome-Wide Analysis of Gene Regulatory Networks of the FVE-HDA6-FLD Complex in Arabidopsis
Source: Front Plant Sci. 2016 Apr 28;7:555. doi: 10.3389/fpls.2016.00555 (PMC4848314; doi:10.3389/fpls.2016.00555)

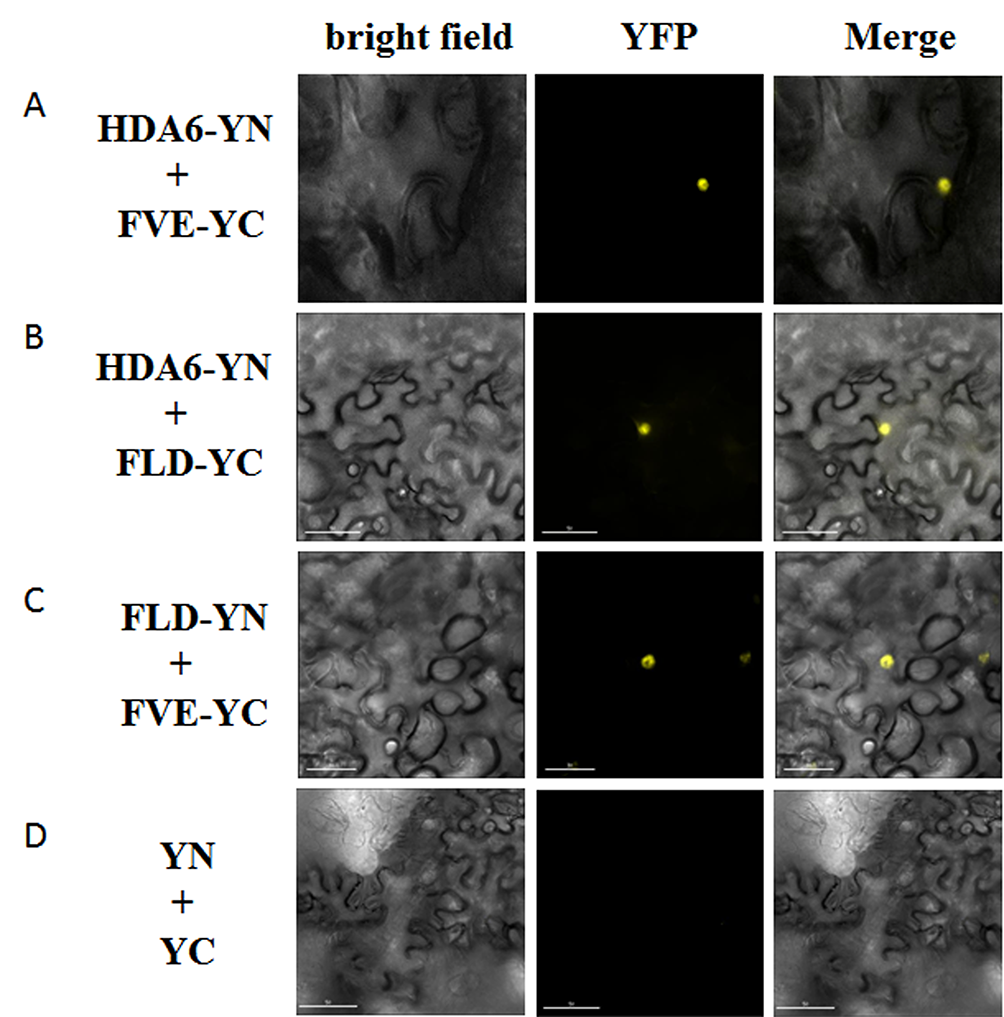

Supplement: Supplementary file 1 [file Image_1.TIF]

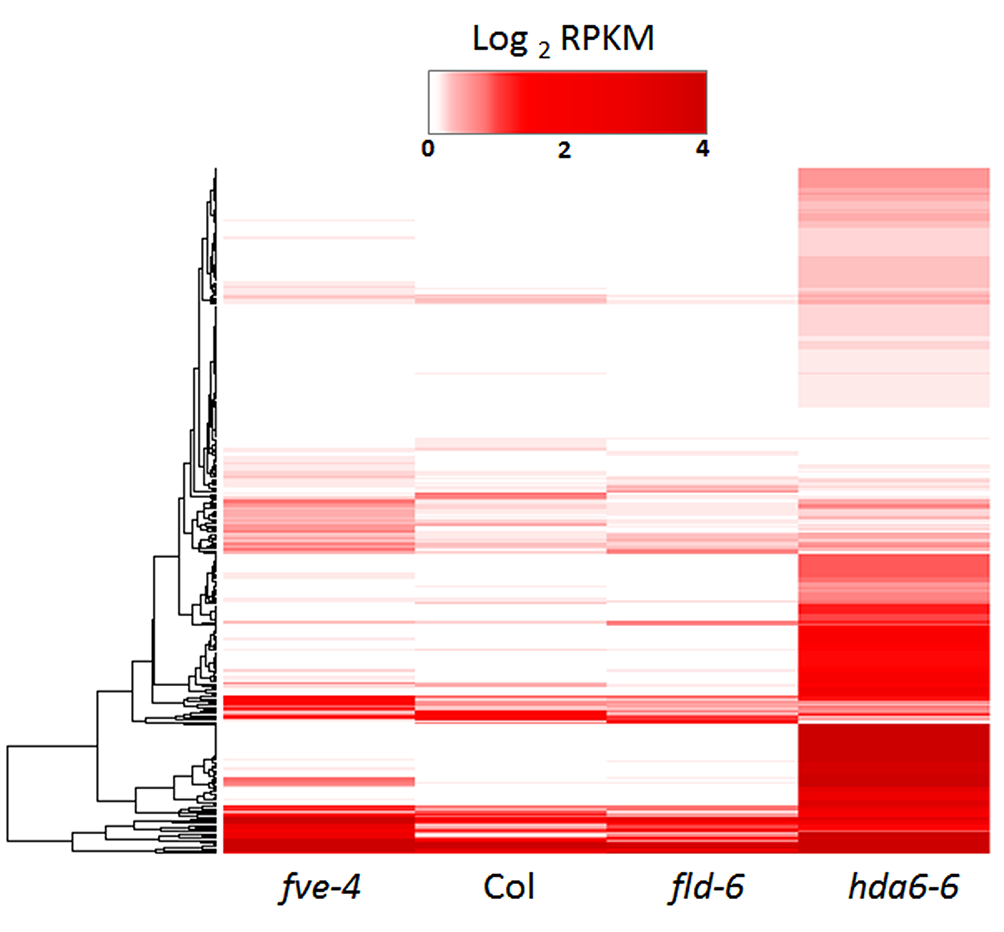

Supplement: Supplementary file 2 [file Image_2.TIF]

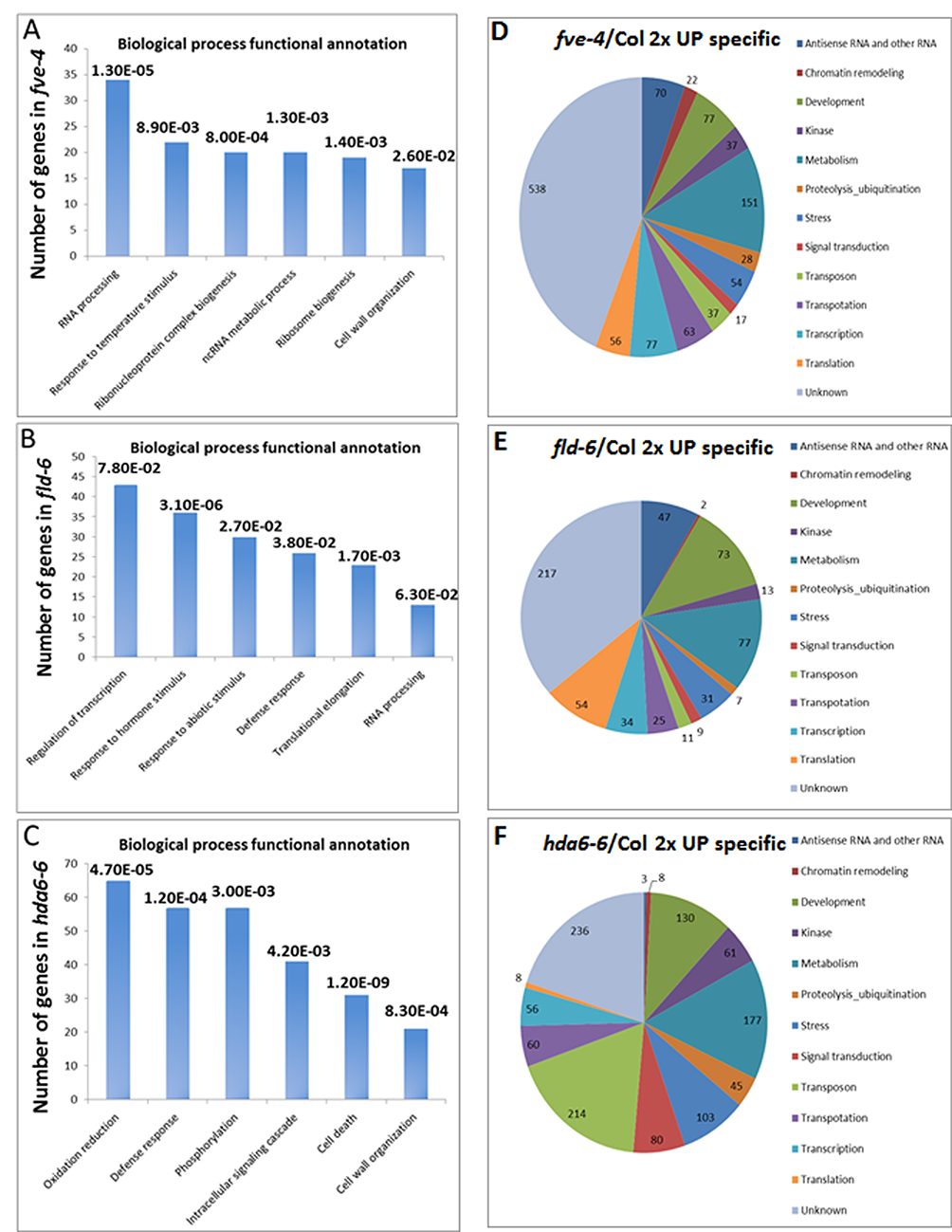

Supplement: Supplementary file 3 [file Image_3.TIF]

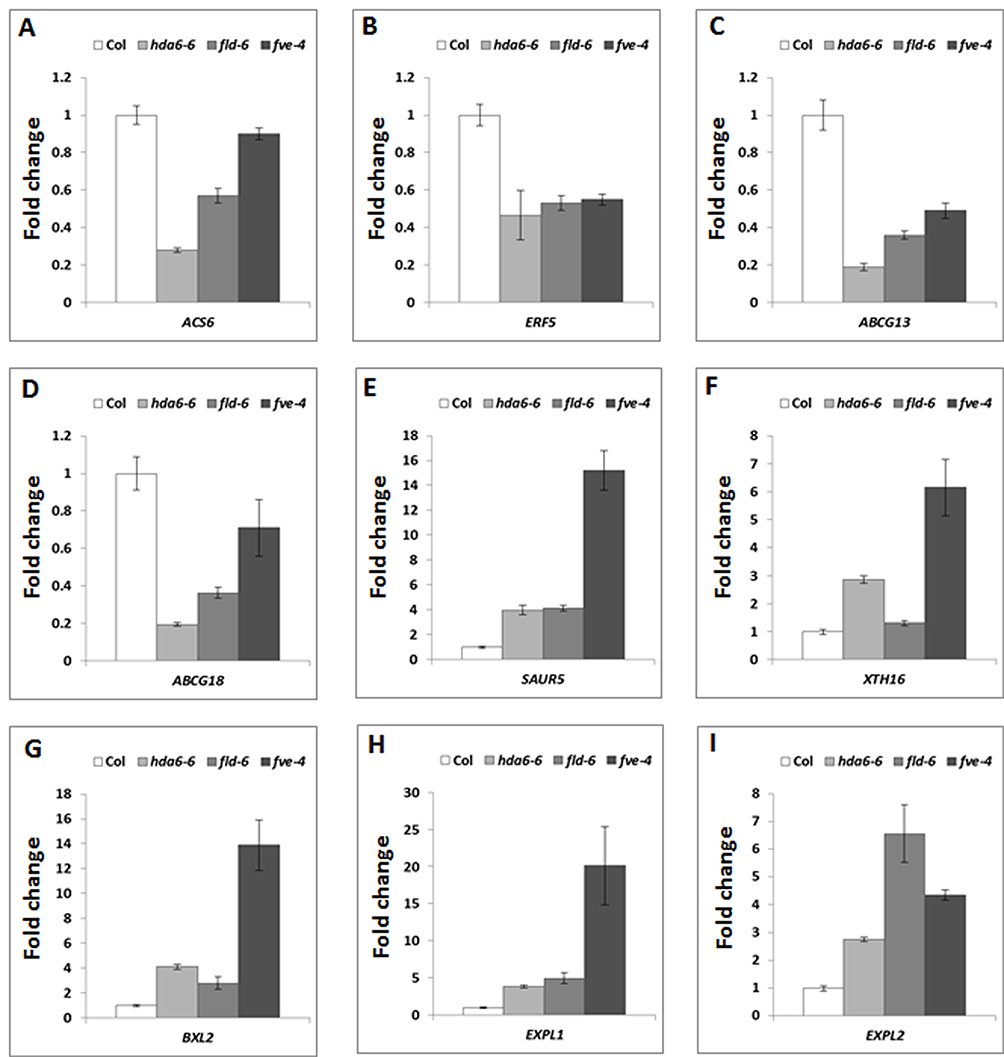

Supplement: Supplementary file 4 [file Image_4.TIF]

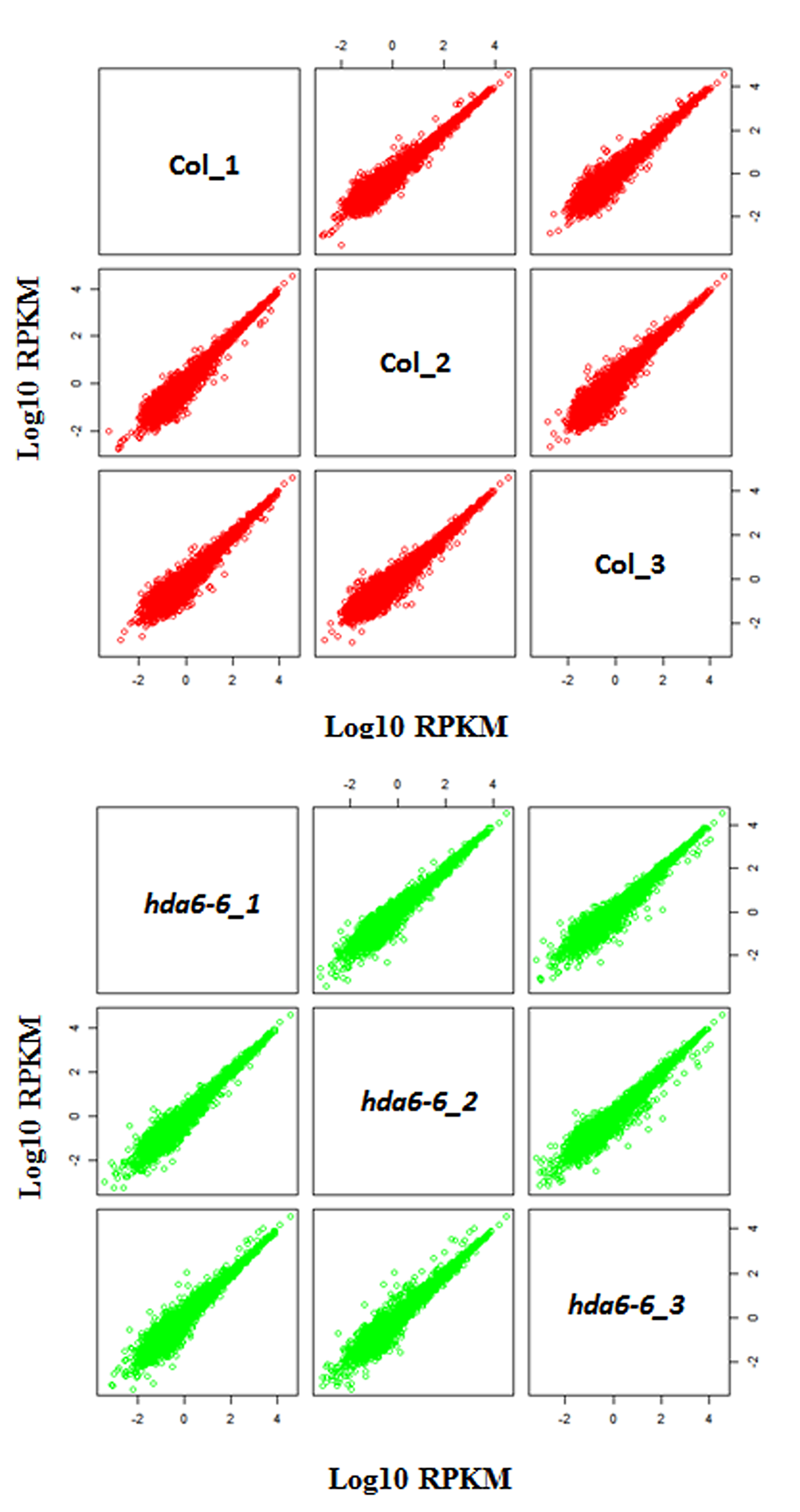

Supplement: Supplementary file 5 [file Image_5.TIF]
