# Supplementary material for: Genome-Wide Analysis of Gene Regulatory Networks of the FVE-HDA6-FLD Complex in Arabidopsis
Source: Front Plant Sci. 2016 Apr 28;7:555. doi: 10.3389/fpls.2016.00555 (PMC4848314; doi:10.3389/fpls.2016.00555)
Supplement: Supplementary file 18 [file Data_Sheet_1.PDF]

Supplementary Material

Genome-wide analysis of gene regulatory networks of the FVE-HDA6-FLD complex in Arabidopsis

Chun-Wei Yu, Kao-Yuan Chang and Keqiang Wu*

*Correspondence: Keqiang Wu: kewu@ntu.edu.tw

Supplementary Figures and Tables

Supplememtal Figure 1. HDA6 and FLD physically interacted with FVE.

Supplememtal Figure 2. Heatmap clustering of transposons up-regulated in *hda6-6*, *fve-4* and *fld-6*.

Supplememtal Figure 3. Functional annotation of genes regulated by FVE, FLD, and HDA6.

Supplememtal Figure 4. Validation of RNA-sequencing results.

Supplememtal Figure 5. Scatterplot of Pearson correlation of three RNA-seq replicates for wild-type (Col) and hda6-6 mutant.

Supplememtal Table 1. Overlap of up-regulated genes found in the *fve-4*, *fld-6*, and *hda6-6* mutants.

Supplememtal Table 2. Overlap of down-regulated genes found in the *fve-4*, *fld-6*, and *hda6-6* mutants.

Supplememtal Table 3. Whole-genome expression profiling comparing the gene expression of Col wild type and *fve-4* mutant grown for 2 weeks under long day conditions. The genes up-regulated $\geq$2 folds were list in the table.

Supplememtal Table 4. Whole-genome expression profiling comparing the gene expression of Col wild type and *fve-4* mutant grown for 2 weeks under long day conditions. The genes down-regulated 2 folds were list in the table.

Supplememtal Table 5. Whole-genome expression profiling comparing the gene expression of Col wild type and *fld-6* mutant grown for 2 weeks under long day conditions. The genes up-regulated $\geq$2 folds were list in the table.

Supplememtal Table 6. Whole-genome expression profiling comparing the gene expression of Col wild type and *fld-6* mutant grown for 2 weeks under long day conditions. The gene down-regulated $\geq$2 folds were list in the table.

Supplememtal Table 7. Whole-genome expression profiling comparing the gene expression of Col wild type and *hda6-6* mutant grown for 2 weeks under

long day conditions. The genes up-regulated $\geq$2 folds and P value $\leq$0.05 were list in the table.

Supplememtal Table 8. Whole-genome expression profiling comparing the gene expression of Col wild type and *hda6-6* mutant grown for 2 weeks under long day conditions. The genes down-regulated $\geq$2 folds and P value $\leq$0.05 were list in the table.

Supplememtal Table 9. Specific genes up-regulated $\geq$2 folds in *fve-4* mutant.

Supplememtal Table 10. Specific genes up-regulated $\geq$2 folds in *fld-6* mutant.

Supplememtal Table 11. Specific genes up-regulated $\geq$2 folds in *hda6-6* mutant.

Supplememtal Table 12. Primers used in this study

Figure legends

Supplememtal Figure 1. HDA6 and FLD physically interacted with FVE.
BiFC assays in tobacco leaves show the interaction between HDA6 and FVE **(A)**, HDA6 and FLD **(B)** or FLD and FVE **(C)** in living cells. HDA6, FVE and FLD were fused with the C terminus (YC) or N terminus (YN) of YFP. Empty vectors (YN and YC) were also co-transfected as the negative control **(D)**.

Supplememtal Figure 2. Heatmap clustering of transposons up-regulated in *hda6-6*, *fve-4* and *fld-6*. Cluster analysis of transposons which were up-regulated in *hda6-6*, *fve-4* and *fld-6*. The transposons represented in at least one of mutants were analyzed. The color bar represents the log2 of the RPKM value.

Supplemental Figure 3. Functional annotation of genes regulated by FVE, FLD, and HDA6. (A-C) Relative number of genes up-regulated in *fve-4*, *fld-6*, and *hda6-6* analyzed by DAVID database. (D-F) Functional classification of genes up-regulated in *fve-4, fld-6,* and *hda6-6*.

Supplemental Figure 4. Validation of RNA-sequencing results.
(A-B) Real-time RT-PCR analysis of the expression of ethylene related genes in Col wild type, *hda6-6, fld-6* and *fve-4* plants grown under LD conditions for 14 days. (C) Gene involved in transport. (D-H) Genes related to cell wall expansion. The values shown are means ±SD.

Supplemental Figure 5. Scatterplot of Pearson correlation of three RNA-seq replicates for wild-type (Col) and *hda6-6* mutant. The coefficients between replicate 1 and 2, 2 and 3, and 1 and 3 are 0.970057, 0.963591, and 0.96409, respectively, for Col

and 0.972537, 0.96653, 0.96772, respectively, for *hda6-6*.
